# Balancing Act: A Comprehensive Review of Vestibular Evaluation in Cochlear Implants

**DOI:** 10.7759/cureus.55261

**Published:** 2024-02-29

**Authors:** Andrea Moreno, Melissa Castillo-Bustamante, Jose A Prieto

**Affiliations:** 1 Otology, Hospital Militar Nueva Granada, Bogotá, COL; 2 Otoneurology, Centro de Vértigo y Mareo, Mexico City, MEX; 3 School of Medicine, Universidad Pontificia Bolivariana, Medellín, COL

**Keywords:** cochlear implant, neuro-otology, otology, vestibular testing, vestibule labyrinth

## Abstract

Cochlear implantation, a transformative intervention for individuals with profound hearing loss, has evolved significantly over the years. However, its impact on the vestibular system, responsible for balance and spatial orientation, remains a subject of ongoing research and clinical consideration. This narrative review highlights key aspects of vestibular evaluation in patients undergoing cochlear implantation. Preoperative vestibular assessment is crucial to establish baseline vestibular function and identify any pre-existing balance issues.

Various tests, including caloric, rotational chair, vestibular-evoked myogenic potential, and video head impulse tests, play a vital role in evaluating vestibular function. The goal is to assess the risk of vestibular disturbances arising from the surgery, guide surgical planning, and detect pre-existing alterations that could be totally or partially compensated.

While some patients experience minimal vestibular disruptions, others may encounter transient or persistent balance issues following cochlear implant surgery. Postoperative vestibular testing allows for the early detection of such disturbances, enabling timely interventions like vestibular rehabilitation and evaluating changes produced due to surgical complications or changes in the patient's prior conditions.

Challenges in vestibular evaluation include individual variability in patient responses, the proximity of the cochlea to the vestibular system, and the need to tailor testing protocols to individual needs. Further research is essential to refine testing protocols, minimize vestibular disturbances, and improve outcomes for cochlear implant candidates. A multidisciplinary approach involving otolaryngologists, audiologists, and physical therapists is integral to comprehensive patient care in this context.

In conclusion, vestibular evaluation in patients undergoing cochlear implantation is critical for optimizing surgical planning, managing postoperative issues, and enhancing the overall quality of life for individuals embarking on the journey of restored hearing.

## Introduction and background

The world of audiology and otology has witnessed remarkable advancements over the past few decades, particularly in the field of cochlear implantation [1]. Cochlear implants have revolutionized the lives of individuals with severe to profound hearing loss, offering them the gift of sound and the opportunity for enhanced communication and quality of life. As these life-changing devices continue to evolve, there is an ever-growing need to address the intricate relationship between hearing and balance within the inner ear [1,2].

The vestibular system, a crucial component nestled deep within the labyrinthine recesses of the inner ear, plays a pivotal role in our daily lives. It not only aids in maintaining equilibrium and posture but also contributes significantly to our spatial awareness and perception of motion [3,4]. While cochlear implantation primarily targets auditory rehabilitation, it is essential to recognize that the inner ear is a complex, interconnected system, and interventions therein may have unintended consequences on the vestibular function of patients [5,6].

This narrative review embarks on a journey through the intricate landscape of vestibular evaluation and testing in the context of cochlear implant recipients. It delves into the multifaceted relationship between the cochlea, the vestibular apparatus, and the central nervous system, shedding light on the challenges and considerations faced by clinicians and researchers in this field.

This narrative review also aims to highlight areas where further research is needed to deepen our understanding of the complex interplay between hearing and balance as well as some specific controversies in this research field. It also seeks to contribute to the ongoing dialogue among clinicians, audiologists, researchers, and patients, thereby ultimately striving for more comprehensive care and improved outcomes for individuals navigating the realm of cochlear implantation.

## Review

This review was conducted between July and October 2023. We used the MeSH headings “cochlear implants" OR “cochlear implantation" AND ("vestibular function test" OR "vestibular system*" OR "vestibule labyrinth*" NOR "vestibular disorders" NOR "central nervous system") to search PubMed, Bvsalud, and Google Scholar databases for relevant articles. The search was limited to articles published between 2013 and 2023 as our goal was to review articles published in the last decade where more studies were released. We included complete manuscripts published in English and articles focused on vestibular testing in candidates for cochlear implants. Studies outside the field of neurotology, otology, and otolaryngology were excluded. Also, articles focused on pediatric populations, articles focused on middle and inner ear malformations, and articles focused on vestibular implants were excluded.

Results were cross-checked among the three authors. In total, 1,220 indexed papers were identified in the initial search. From these, only 21 indexed articles were selected because they reported patients undergoing cochlear implants with vestibular testing.

**Figure 1:**
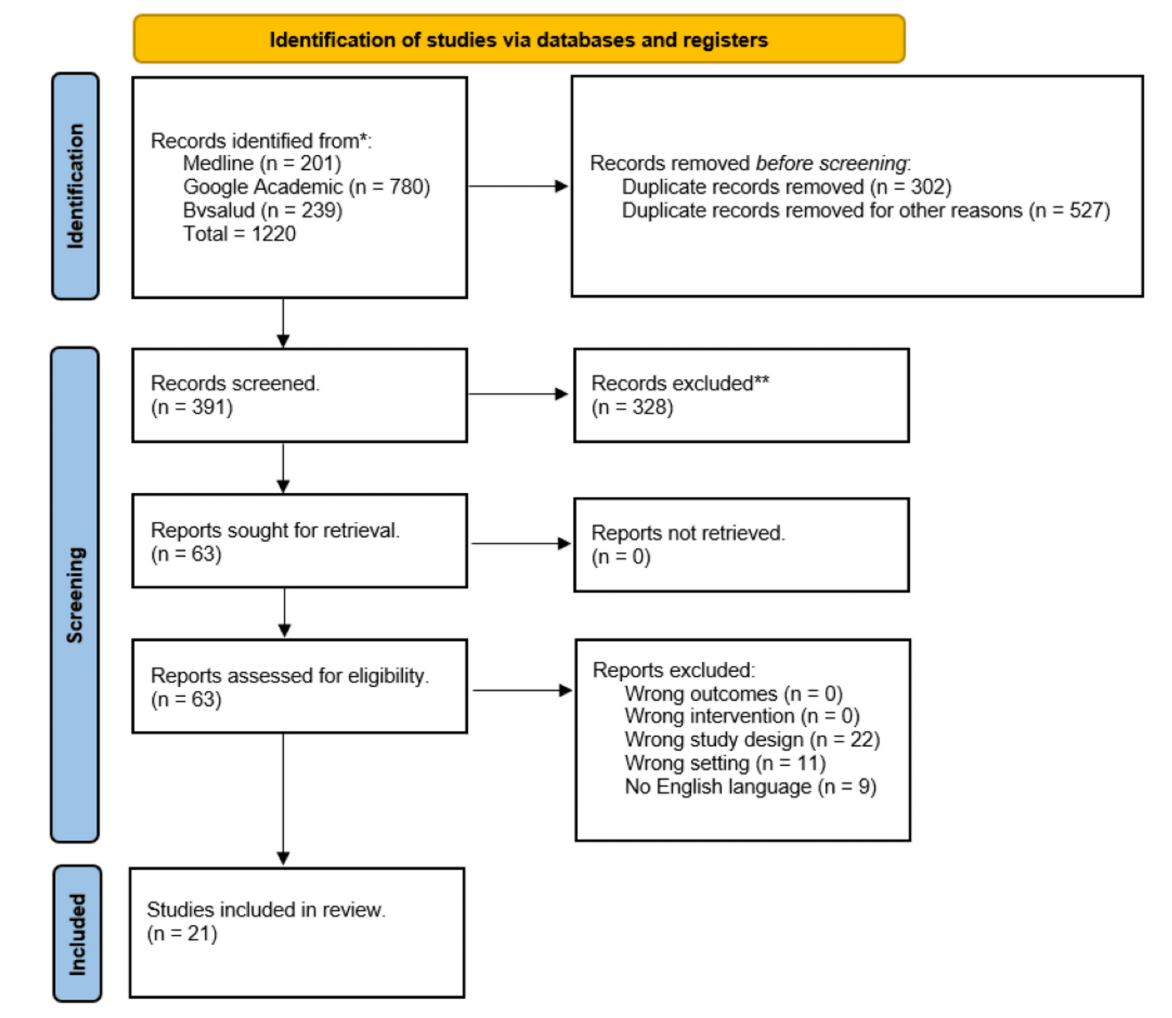
Study flowchart *Records were identified from MEDLINE, Google Academic, and Bvsalud. All authors conducted this search, and the results were cross-checked. **Records with incomplete articles, abstracts, preprints, and non-English language articles were excluded.

The quality of evidence in the published articles was reviewed according to the 2009 Levels of Evidence of the Oxford Centre for Evidence-Based Medicine (Figure 1).

Pre-implant vestibular evaluation

Before embarking on cochlear implant surgery, a comprehensive evaluation of the patient's vestibular system is imperative. The vestibular system plays a main role in regulating balance and spatial orientation, and any anomalies in this system can have a direct influence on the outcomes following implantation [7]. Vestibular testing assumes paramount importance due to the intimate anatomical relationship between the cochlea, responsible for auditory function, and the vestibular system, responsible for balance control [8]. Surgical intervention, as underscored in previous studies [7,8], has the potential to disrupt this delicate equilibrium.

Conducting a preoperative assessment is of utmost significance as it aids in the detection of any pre-existing vestibular disorders or equilibrium-related concerns [9]. The identification of such conditions is instrumental in anticipating their potential impact on the patient's postoperative journey and their subsequent rehabilitation requirements [9].

The evaluation typically starts with a thorough clinical history and physical examination, focusing on any pre-existing balance or dizziness issues [10,11]. One of the primary objectives of pre-implant vestibular evaluation is to establish a baseline of the patient's vestibular function. This baseline serves as a reference point for comparing postoperative changes [10,11].

A pre-implant vestibular assessment can be crucial in the selection of a suitable ear for implantation, particularly when faced with a situation where both ears exhibit profound hearing loss, yet one ear demonstrates superior vestibular function [12]. This decision aims to minimize the risk of vestibular disturbances. Additionally, the results of vestibular testing may offer valuable insights for the surgical team, enabling them to consider a specific electrode array design or surgical technique that can mitigate the impact on the vestibular system [12,13]. Another aspect to consider is the estimation of vestibular damage risk [14]. This assessment plays a critical role in gauging the likelihood of patients encountering balance-related challenges after the surgical procedure [14]. Individuals exhibiting substantial vestibular deficits may necessitate a more prudent and cautious surgical approach [14]. Multiple vestibular tests are conducted as part of the preoperative assessment (Table 1).

**Table 1:** Vestibular testing described in patients undergoing cochlear implant

Tests	Rationale
Videonystagmography (VNG) [14]	This test entails monitoring eye movements in reaction to visual stimuli and head motions, aiding in the diagnosis of a range of vestibular disorders.
Caloric test [15]	This test assesses the function of the semicircular canals by irrigating the external ear canal with warm and cold water. The resulting eye movements (nystagmus) help determine if one or both ears are affected.
Rotational chair test [16]	Patients sit in a motorized chair that rotates at different speeds. This assesses the function of the semicircular canals and the vestibulo-ocular reflex.
Video head impulse test (vHIT) [17]	It measures the vestibulo-ocular reflex (VOR) by tracking eye movements while the head is rapidly moved in various directions. These movements are rapid and unpredictable head movements while observing a fixed target and are made on the horizontal and vertical planes.
Cervical vestibular-evoked myogenic potentials (c-VEMPs) and ocular vestibular-evoked myogenic potentials [18,19]	Vestibular-evoked myogenic potentials (VEMPs) are short-latency, mainly otolith-driven vestibular reflexes elicited by air-conducted sound (ACS), bone-conducted vibration (BCV), or galvanic vestibular stimulation and recorded from the inferior oblique eye muscle (ocular or oVEMPs) or the sternocleidomastoid muscle (cervical or c-VEMPs). While the oVEMP n10p16 response predominantly represents a contralateral dynamic utricular function, the cVEMP p13n23 amplitude is predominantly an indicator of ipsilateral saccular function.
Posturography [20,21]	Computerized dynamic posturography uses a force platform that can both translate and rotate. When combined with visual stimuli, it can be used to determine the relative importance of the various sensory inputs critical for balance, namely vision, somatosensation, and vestibular sensation.

Vestibular testing in the context of cochlear implantation has been the subject of significant research and documentation in the medical literature [14-21]. Various methods have been employed to assess vestibular function in these patients [15-21]. These include caloric testing, rotational chair testing, video head impulse testing (vHIT), and vestibular-evoked myogenic potential (VEMP) assessments [15-21]. Caloric testing involves assessing responses to warm- and cold-water irrigations into the ear canal [14,15]. vHIT measures the high-frequency head movements and eye responses to detect subtle abnormalities [18], and VEMP assesses the otolith organs' function [17,18]. These tests collectively provide valuable insights into vestibular status following cochlear implantation and are reported in the literature to aid in the diagnosis, management, and rehabilitation of vestibular issues, contributing to improved patient outcomes and care. Studies and facts are shown in Tables 2, 3, 4 [22-28].

**Table 2:** Video head impulse test findings

Author	Number of patients	Type of the study	Facts
Bittar et al., 2019 [22]	31	Cross-sectional	Gain > 0.8; abnormal values only in subjects with vertigo as compared to the preoperative video head impulse test results.
Bittar et al., 2019 [23]	57	Cross-sectional	Gain less than 0.8; canal hypofunction 36.8% of patients with abnormal video head impulse test before the cochlear implant procedure.
Rasmussen et al., 2021 [24]	35	Prospective study	The mean gain in implanted ears decreased insignificantly from 0.93 to 0.89.
West et al., 2021 [25]	40	Prospective study	Mean video head impulse test gain decreased from preoperative 0.92 to postoperative 0.84.
Jutila et al., 2013 [26]	44	Prospective study	Gain on the operated side was 0.77 ± 0.26 preoperatively, 0.75 ± 0.30 in the early, and 0.73 ± 0.33 in the late postoperative control and did not change significantly. Mean asymmetry remained within 9%-10% on all test occasions.

**Table 3:** Caloric testing results

Author	Number of patients	Type of study	Facts
Parietti-Winkler et al., 2021 [7]	10	Prospective study	From the Jongkees formula, an inter-ear difference > 20%, hyporeflexia, and areflexia were mostly reported in patients undergoing cochlear implants before and after the procedure.
Bittar et al., 2019 [23]	57	Cross-sectional cohort	Vestibular hypofunction or canal paresis in patients before the procedure.
Rasmussen et al., 2021 [24]	35	Prospective study	From the Jongkees formula, side differences > 25% were considered altered. Mean unilateral weakness increased from 19% to 40% from T0 to T2 (p < 0.005), and the total number of patients with either hypofunction or areflexic semicircular canals increased significantly from 7 to 17 (p < 0.005).
West et al., 2021 [25]	40	Prospective study	From the Jongkees formula, side differences > 25% were considered altered. Mean caloric unilateral weakness increased from 20.5% preoperative to 42.9% postoperative (p < 0.0001).
Batuecas-Caletrio et al., 2015 [27]	30	Prospective study	From the Jongkees formula, side differences > 25% were considered altered. Differences > 10% between postoperative response and preoperative tests were considered abnormal.
Piker et al., 2020 [28]	10	Cohort	From the Jongkees formula, side differences > 25% were considered altered.

**Table 4:** Presurgical and postsurgical changes of cervical vestibular-evoked myogenic potentials (c-VEMPs) and ocular vestibular-evoked myogenic potentials (o-VEMPs)

Author	Number of patients	Type of study	Facts
West et al., 2021 [25]	40	Prospective study	The difference between primarily binary (response) and secondarily amplitude size > 20 mV was abnormal in the cVEMP.
Miwa et al., 2019 [29]	9	Prospective study	Before surgery, cervical vestibular-evoked myogenic potential responses were diminished in five of the nine patients (55.6%), while the results of stabilometry were poor in six patients (66.7%). After surgery, both cervical vestibular-evoked myogenic potential response findings in the cochlear implant mode exhibited significant deterioration relative to the preoperative results (cervical vestibular-evoked myogenic potentials 7/9, 77.8%).
Melvin et al., 2009 [30]	36	Prospective study	Reduced saccular function by cervical vestibular-evoked myogenic potentials present preoperatively and absent postoperatively or with an increase in threshold > 10 dB postoperatively.

Challenges and facts

Vestibular testing in patients considered for cochlear implantation can pose several challenges [25-29]. These challenges can arise due to the intricate interplay between the auditory and vestibular systems as well as the unique considerations associated with cochlear implant surgery.

First and foremost, cochlear implant surgery has the potential to affect the delicate structures within the inner ear, which could result in vestibular disturbances such as dizziness or imbalance [29,31-35]. These complications underscore the paramount importance of a comprehensive vestibular assessment as an integral component of post-implant care [32,35].

One particular challenge stems from the variability in patient experiences as not all recipients of cochlear implants will manifest vestibular symptoms [8]. Consequently, the identification of individuals at risk of vestibular issues can be intricate, necessitating customized assessments and a vigilant approach to monitoring [8]. Another fact to consider is the importance of early detection and intervention. Timely vestibular assessment can help healthcare providers address emerging problems promptly, mitigating potential falls and optimizing the rehabilitation process [8,32].

Lastly, ongoing research and advancements in vestibular testing techniques, such as video head impulse testing and vestibular-evoked myogenic potentials, are continuously enhancing our ability to diagnose and manage vestibular issues in this patient population [32]. Vestibular testing in cochlear implant patients is a multifaceted endeavor, marked by both challenges and promising facts that contribute to improved care and outcomes for individuals with hearing impairment undergoing these transformative procedures [24-37]. Challenges and facts described in the literature are shown in Table 5.

**Table 5:** Challenges and facts of vestibular assessment in patients undergoing cochlear implants

Challenges	Facts
Individual variability [8]	Patients' responses to cochlear implant surgery can vary widely. Some individuals may experience significant vestibular disturbances, while others may have minimal or no issues. Predicting how an individual will respond can be challenging.
Pre-existing vestibular Issues [24]	Patients may already have vestibular problems, such as balance disorders or inner ear conditions, before considering cochlear implants. Distinguishing between pre-existing issues and those potentially caused by the surgery can be challenging.
Impact of surgery [32]	Cochlear implant surgery involves inserting electrodes into the cochlea, which is situated within the inner ear, near the vestibular system. This proximity increases the risk of unintentional vestibular system disruption during surgery.
Choice of vestibular tests [33]	Selecting the appropriate vestibular tests for each patient and determining the timing of these tests (preoperative, postoperative, or both) can be complex. To date, there is no one-size-fits-all approach.
Interpretation of results [33]	Interpreting vestibular test results can be challenging, especially when subtle changes are observed. Distinguishing between temporary and permanent vestibular issues can be difficult. Further training is needed.
Patient factors [34]	Patient cooperation is crucial for accurate testing, especially in tests that involve head movement or changes in body position. Young children or patients with cognitive impairments may find it challenging to participate in these tests effectively.
Surgical technique [35]	The surgical technique used during cochlear implantation can influence the risk of vestibular disruption. Surgeons are suggested to optimize surgical approaches to avoid or minimize trauma to the delicate structures of the inner ear.
Rehabilitation [37]	Vestibular rehabilitation programs were sometimes recommended for patients experiencing post-implantation balance issues. These programs aim to help patients adapt to any vestibular changes and improve their balance. Post-surgery, the emergence of vestibular issues may necessitate the development and execution of tailored rehabilitation strategies, a task that can pose significant challenges. Vestibular rehabilitation often demands a multidisciplinary approach, involving collaboration among audiologists, otolaryngologists, and physical therapists.
Ethical considerations [38]	Balancing the potential benefits of cochlear implantation with the risk of vestibular disturbances raises ethical considerations. Patients should be fully informed of potential risks before making treatment decisions.
Research gaps [39]	Further research is essential to gain a deeper understanding of the relationship between cochlear implantation and vestibular function. This encompasses the identification of risk factors, refining testing protocols, and the formulation of strategies aimed at minimizing disturbances to the vestibular system.

Elections and key issues

Within the healthcare domain, elections assume a critical role in shaping policies and priorities. This includes the consideration of key issues concerning patients undergoing cochlear implantation and requiring vestibular assessments [31-40]. During election periods, it becomes imperative for candidates and policymakers to acknowledge and address the distinctive challenges encountered by these patients [31-40]. One of the primary concerns is ensuring equitable access to cochlear implantation services and comprehensive vestibular assessments for all individuals, regardless of their socioeconomic status or geographic location [31-40]. Furthermore, candidates must focus on promoting research and innovation in the field to enhance the effectiveness and safety of cochlear implant procedures while also addressing any potential reimbursement issues for patients [31-40]. By emphasizing these key issues in healthcare elections, we can strive to improve the quality of life and accessibility to essential services for individuals with hearing impairments who require vestibular assessment alongside cochlear implantation. Key issues and facts are listed in Table 6.

**Table 6:** Key issues and facts of vestibular assessment in patients undergoing cochlear implants

Key issues	Facts
Standardization of protocol [8]	There was a lack of standardized protocols for vestibular testing in cochlear implant candidates. The choice of tests, timing of testing (preoperative, postoperative, or both), and criteria for assessing vestibular function varied among healthcare institutions and providers.
Patient consent and informed decision-making [10]	Some experts emphasized the importance of ensuring that patients had a thorough understanding of the potential risks and benefits of vestibular testing as part of the informed consent process.
Routine vs. selective testing [25]	To date, there is no consensus on whether vestibular testing should be routinely conducted in all cochlear implant candidates or whether it should be selectively administered based on individual factors such as age, medical history, and the presence of pre-existing balance issues.
Individual variation [31]	Individual responses to cochlear implantation and its effects on vestibular function varied widely. Factors such as electrode array design, surgical technique, and the specific cochlear implant model used could influence outcomes.
Interpretation of results [37]	Interpreting vestibular test results, especially in the context of cochlear implantation, could be challenging. There are controversies about whether subtle changes in vestibular function detected by testing were clinically significant or merely artifacts of the testing process.
Clinical utility [38]	Some clinicians questioned the clinical utility of routine vestibular testing, especially when it did not necessarily change the surgical plan or postoperative management for many patients. Critics argued that the potential benefits of such testing did not always outweigh the costs and potential patient discomfort.
Mixed findings [40]	Research outcomes were mixed, with some studies indicating minimal impact on vestibular function following cochlear implant surgery, while others reported transient or even persistent vestibular issues.
Impact of testing on patient anxiety [41]	For some patients, undergoing vestibular testing, particularly when not deemed essential, could cause anxiety and discomfort. This raised ethical questions about whether the potential benefits of testing justified the psychological impact on patients.
Lack of research [42]	The evidence base for the impact of cochlear implantation on vestibular function and the effectiveness of vestibular testing protocols was still evolving. More research was needed to establish best practices.

Considerations on postoperative vestibular evaluation

After cochlear implantation, postoperative balance assessments are conducted to monitor any changes in vestibular function [8,40]. This facilitates prompt intervention in case of any balance-related concerns [8,40]. Vestibular testing typically forms a component of a comprehensive evaluation process, which entails collaboration among a multidisciplinary team comprising otolaryngologists, audiologists, and physical therapists [8,40].

Conducting a postoperative vestibular evaluation enables the timely identification of any vestibular disruptions or balance problems resulting from the surgical procedure [40,42]. In certain instances, vestibular issues may be linked to the settings of the cochlear implant [40,42]. Postoperative testing can guide audiologists and otolaryngologists in fine-tuning these settings to minimize disturbances [40-42].

Vestibular function can evolve, and postoperative testing provides a means to monitor these changes [42,43]. This is especially important for patients who experience temporary vestibular issues that may improve with time [42,43]. If vestibular disturbances persist, vestibular rehabilitation programs can be tailored to the patient's needs based on postoperative evaluation results [42,43]. These programs help patients adapt to any vestibular changes and improve their balance [40,43].

Some patients may require periodic postoperative vestibular assessments to track changes and ensure ongoing care as needed [8,30,40]. Post-cochlear implant vestibular testing contributes to research efforts aimed at reducing the impact of cochlear implantation on the vestibular system through improved surgical techniques and electrode designs [8,30,40].

Limitations

Vestibular testing before and after cochlear implantation is essential to ensure the comprehensive care and well-being of patients [8,14,25]. First, it is imperative to establish standardized protocols for post-implant vestibular assessments, including assessments of balance, spatial orientation, and gaze stability [8,14,25]. Additionally, healthcare providers should prioritize regular follow-up appointments to monitor and address any vestibular complications or changes in patients' conditions [44]. Furthermore, investing in advanced technology and diagnostic tools can enhance the accuracy and efficiency of these assessments [44]. Lastly, incorporating vestibular rehabilitation programs into the post-implant care plan can help patients adapt to any vestibular deficits and improve their overall quality of life [43,45]. These proposals aim to optimize the long-term outcomes and safety of individuals who have undergone cochlear implantation while considering their vestibular health.

Cochlear implantation should also encompass fall risk assessment and evaluation of quality of life [46]. To achieve a comprehensive approach to patient care, healthcare providers should incorporate fall risk assessments as a routine component of post-implant vestibular testing [46]. This includes evaluating factors such as gait stability, postural control, and proprioception to identify patients at higher risk of falls. Interventions like vestibular rehabilitation can then be tailored to address these risks and enhance patients' safety [46].

Simultaneously, assessing the impact of cochlear implantation and vestibular health on patients' quality of life is crucial [47]. By utilizing validated quality-of-life questionnaires specific to hearing-impaired individuals, healthcare providers can gain insights into how vestibular status influences daily functioning, social interactions, and emotional well-being [47]. This evaluation can guide personalized care plans, ensuring that patients receive not only effective medical treatment but also support for their holistic well-being [47]. By integrating fall risk assessments and quality of life evaluations into post-implant vestibular testing protocols, we can offer more comprehensive and patient-centered care to those with cochlear implants [46].

Vestibular testing and assessment in patients undergoing cochlear implantation, while crucial for their overall well-being, come with certain limitations [48]. One significant limitation is the potential discomfort and anxiety experienced by patients during the assessment, especially if they are already dealing with hearing loss and the stress of the cochlear implantation procedure [48]. This psychological factor can influence the accuracy of results [48]. Additionally, vestibular testing tools and techniques may not always capture subtle or intermittent vestibular dysfunction, leading to possible underdiagnosis of issues. Furthermore, some patients may have pre-existing vestibular conditions that complicate the interpretation of post-implant assessments [46-48]. The availability and accessibility of specialized vestibular testing equipment and expertise can also be limited in certain healthcare settings, potentially resulting in delayed or incomplete assessments [49]. In conclusion, while vestibular testing is a valuable component of post-cochlear implant care, it is important to recognize and address these limitations to provide the most comprehensive and patient-centered care possible for individuals undergoing this life-changing procedure.

## Conclusions

In conclusion, the evolution of cochlear implantation as a transformative intervention for individuals with profound hearing loss underscores the importance of comprehensive vestibular evaluation in optimizing surgical outcomes and managing postoperative issues. The preoperative assessment establishes baseline vestibular function and identifies potential balance issues, while various tests aid in evaluating vestibular function and guiding surgical planning. Although some patients may experience minimal vestibular disruptions, others may encounter transient or persistent balance issues following surgery, highlighting the need for postoperative testing and timely interventions like vestibular rehabilitation.

Challenges in the vestibular evaluation include individual variability in patient responses and the proximity of the cochlea to the vestibular system, necessitating tailored testing protocols and a multidisciplinary approach involving otolaryngologists, audiologists, and physical therapists. Further research is crucial to refine testing protocols, minimize vestibular disturbances, and improve outcomes for cochlear implant candidates. Overall, vestibular evaluation plays a critical role in enhancing the overall quality of life for individuals undergoing cochlear implantation by optimizing surgical planning and managing postoperative issues effectively.
