# Correction: Balancing Act: A Comprehensive Review of Vestibular Evaluation in Cochlear Implants

**DOI:** 10.7759/cureus.c347

**Published:** 2025-10-03

**Authors:** Andrea Moreno, Melissa Castillo-Bustamante, Jose A Prieto

**Affiliations:** 1 Otology, Hospital Militar Nueva Granada, Bogotá, COL; 2 Otoneurology, Centro de Vértigo y Mareo, Mexico City, MEX; 3 School of Medicine, Universidad Pontificia Bolivariana, Medellín, COL

This article has been corrected to fix the following typo in the second paragraph of the Review section: "In total, 391 indexed papers were identified in the initial search." has been corrected to "In total, 1,220 indexed papers were identified in the initial search."

